# Analysis of Salinity Tolerance in Tomato Introgression Lines Based on Morpho-Physiological and Molecular Traits

**DOI:** 10.3390/plants10122594

**Published:** 2021-11-26

**Authors:** Ahmed Abdelrahim Mohamed Ali, Walid Ben Romdhane, Mohamed Tarroum, Mohammed Al-Dakhil, Abdullah Al-Doss, Abdullah A. Alsadon, Afif Hassairi

**Affiliations:** 1 Plant Production Department, College of Food and Agricultural Sciences, King Saud University, P.O. Box 2460, Riyadh 11451, Saudi Arabia; asaif@ksu.edu.sa (A.A.M.A.); walid.brm3@gmail.com (W.B.R.); maaldakhil@Kacst.edu.sa (M.A.-D.); aaldoss@ksu.edu.sa (A.A.-D.); alsadon@ksu.edu.sa (A.A.A.); 2 Department of Botany and Microbiology, College of Science, King Saud University, P.O. Box 11451, Riyadh 11451, Saudi Arabia; mtarroum@ksu.edu.sa; 3 Natural Resources and Environmental Research Institute, King Abdulaziz City for Science and Technology, Riyadh 11442, Saudi Arabia; 4 Centre of Biotechnology of Sfax, University of Sfax, B.P 1177, Sfax 3018, Tunisia

**Keywords:** *Solanum habrochaites*, *Solanum pennellii*, ILs, tomato, salt stress, salt-stress related genes

## Abstract

The development of salt-tolerant tomato genotypes is a basic requirement to overcome the challenges of tomato production under salinity in the field or soil-free farming. Two groups of eight tomato introgression lines (ILs) each, were evaluated for salinity tolerance. Group-I and the group-II resulted from the following crosses respectively: *Solanum lycopersicum* cv-6203 × *Solanum habrochaites* and *Solanum lycopersicum* M82 × *Solanum pennellii*. Salt tolerance level was assessed based on a germination percentage under NaCl (0, 75, 100 mM) and in the vegetative stage using a hydroponic growing system (0, 120 mM NaCl). One line from group I (TA1648) and three lines from group II (IL2-1, IL2-3, and IL8-3) were shown to be salt-tolerant since their germination percentages were significantly higher at 75 and 100 mM NaCl than that of their respective cultivated parents cvE6203 and cvM82. Using the hydroponic system, IL TA1648 and IL 2-3 showed the highest value of plant growth traits and chlorophyll concentration. The expression level of eight salt-responsive genes in the leaves and roots of salt-tolerant ILs (TA1648 and IL 2-3) was estimated. Interestingly, *SlSOS1*, *SlNHX2*, *SlNHX4*, and *SlERF4* genes were upregulated in leaves of both TA1648 and IL 2-3 genotypes under NaCl stress. While *SlHKT1.1*, *SlNHX2*, *SlNHX4*, and *SlERF4* genes were upregulated under salt stress in the roots of both TA1648 and IL 2-3 genotypes. Furthermore, *SlSOS2* and *SlSOS3* genes were upregulated in TA1648 root and downregulated in IL 2-3. On the contrary, *SlSOS1* and *SlHKT1.2* genes were upregulated in the IL 2-3 root and downregulated in the TA1648 root. Monitoring of ILs revealed that some of them have inherited salt tolerance from *S. habrochaites* and *S. pennellii* genetic background. These ILs can be used in tomato breeding programs to develop salt-tolerant tomatoes or as rootstocks in grafting techniques under saline irrigation conditions.

## 1. Introduction

Salinity is a prevalent problem and a substantive threat to crop production since it attacks approximately 20% and 33% of the total arable and irrigated cultivated area worldwide, respectively [1]. This problem is more common in arid and semi-arid climates due to the low rainfall rate, high temperature during the summer, and low quality of groundwater. Salinity causes osmotic stress, ion imbalance, and ion toxicity which leads to adverse effects on many physiological and biochemical aspects of plants at all developing stages, which seriously affect plant productivity [2]. For these reasons, significant efforts have been devoted to investigate molecular and physiological mechanisms of salinity tolerance in plants and to assist plant breeders for achieving enhanced crop tolerance to salt stress. Despite this substantial effort, only a small number of cultivars, partially tolerant to salinity, were developed due to the complexity of plant response to salinity [3].

Tomato (*Solanum lycopersium* Mill.) is an annual herbaceous plant and a member of the Solanaceae family. Cultivated tomatoes are classified as moderately sensitive to salinity. Mining for salt tolerance potential in tomato wild relative species started in 1941 by Lyon [4]. The genetic variability for salt tolerance traits is limited in domesticated tomatoes, while the wild *Solanum* species have been reported as a source of salt tolerance such as *Solanum pimpinellifolium*, *Solanum Pennellii* (SP), *Solanum cheesmaniae*, *Solanum habrochaites* (*Lycopersicon hirsutum*), *Solanum chmielewskii*, and *Solanum peruvianum* [5]. During domestication, abiotic stress tolerance has been lost in cultivated tomatoes. 

The development of tomato salt-tolerant cultivars by the introgression of salt tolerance traits from wild species is an attractive solution to alleviate the salinity problem [6]. A mapping population of tomatoes was developed from the crossing between the wild species *S. pennellii* (LA0716) and *S. lycopersicum* cultivar M82 (LA3475) [7]. The first offspring were backcrossed to M82 for three generations and then followed by self-pollination for eight generations. As a result of the current work, a set of 50 ILs (introgression lines) were obtained. The ILs are nearly isogenic to M82 with a single homozygous segment of *S. pennellii* chromosome, and therefore all the genetic variation that characterizes them can be associated with the introgressed segment [8]. As early as 2000, other advanced mapping population for tomatoes was developed by Monforte and Tanksley [9]. The novel population was generated from a cross between wild accession *S. habrochaites* and cultivated tomato cv. E6203. This population consisted of a set of 99 near-isogenic lines (NILs) and backcross recombinant inbred lines (BCRILs) with coverage of more than 85% of the *S. habrochaites* genome represented into each line by a single defined introgression [9].

In order to select efficient salt-tolerant tomato ILs, the evaluation must be performed at different growth stages using qualitative and quantitative parameters at a physiological and molecular level. In addition, monitoring the relative expression level of salt stress-related genes by qPCR is an essential step for identifying salt-tolerant ILs [10,11]. Indeed, plants respond to abiotic stresses at the molecular level by expressing several genes whose products are thought to function not only as effector proteins conferring stress tolerance but also as transcription factors and as signal transduction in stress response [2,12]. 

Salt tolerance a complex trait is controlled by several gene families through genetic regulatory networks, which switch on/off the gene expression according to signal responses to the environmental condition surrounding the plant [13]. Many studies carried out to date have provided valuable information about the salt tolerance key genes and mechanisms inside the plant cells. The most studied genes are those coding for osmoprotectant proteins such as proline, glycine betaine, and sugars [14]. In addition, other mechanisms focused on maintaining the concentration of Na^+^ ions against K^+^ and Ca2^+^ ions at the most advantageous level in the cell through active and diffusion mechanisms by ion channels and ion transporters [15]. Ion-detoxification mainly depends on three ion transporters: Salt overly sensitive (SOS), high-affinity K^+^ transporter (HKTs), and Na^+^/H^+^ Exchange (NHXs) transporters. The products of these genes can collaborate to remove Na^+^ from cytosol, transport Na^+^ from root cells to xylem, and achieve ion compartmentation in the vacuoles [16,17]. The SOS pathway is regulated by the interaction between Ca2^+^ and calcium-binding protein (SOS3), which activates a serine/threonine-protein kinase (SOS2) and then together they regulate the activation of a plasma membrane Na^+^/H^+^ antiporter (SOS1) involved in Na^+^ extrusion [18,19]. The first description for high-affinity K^+^ (HKT) gene family in wheat was reported by Schachtman and Schroeder [20]. In addition to its importance for plant nutrient uptake, especially under K^+^ deficiency, HKT contributes to salinity stress tolerance. The HKT transporters family (class I), which is mainly localized to the xylem/symplast boundary of roots and shoots in monocots and dicots show specificity for Na^+^ [21], while the HKT class II has been shown to have a role in Na^+^ and K^+^ transport exclusively in monocots [22]. In contrast to SOS antiporters, cation/proton antiporters on plasma membrane and vacuole, generally called NHX, are ones of the most important antiporters families in the plant. It plays a crucial role under salinity stress in deposition of excess Na^+^ in vacuoles, regulates the homeostasis of K^+^, and adjusts the pH inside the cell [23,24]. The ethylene responsive factor (AP2/ERF) is a major transcription factors in plant, which mainly regulates ethylene signaling. It is also known to be involved in plant development, hormone signal transduction, metabolite regulation, and stress responses [25]. 

One of the strategies in a tomato breeding program that aim to increase tolerance to biotic and abiotic stresses is to use native germplasm or wild relatives for the introgression of new allelic combinations in the current varieties. In this study, we evaluated the salt tolerance level of 16 ILs obtained from the Genetics Resource Centre (TGRC, University of California, Davis, Department of Plant Sciences, USA) in order to use the performing lines as rootstocks under salinity conditions in the field or in soilless agriculture. The 16 ILs derived from the following crosses: Group-I (*Solanum lycopersicum* cv-6203 × *Solanum habrochaites*) and group-II (*Solanum lycopersicum* M82 × *SolanumPennellii*). Indeed, the ILs containing genomic background from *Solanum habrochaites* or *SolanumPennellii* were reported to show certain levels of salt tolerance. We identified two candidate salt-tolerant ILs based on tomato morphological traits, chlorophyll, Na^+^, and K^+^ concentration, and gene expression level by quantification of transcripts accumulation using qRT-PCR. Furthermore, these ILs can be used as rootstocks in grafting experiment under saline irrigation conditions.

## 2. Results

### 2.1. Evaluation of Salt Tolerance of Tomato ILs at Germination Stage

**Figure 1:**
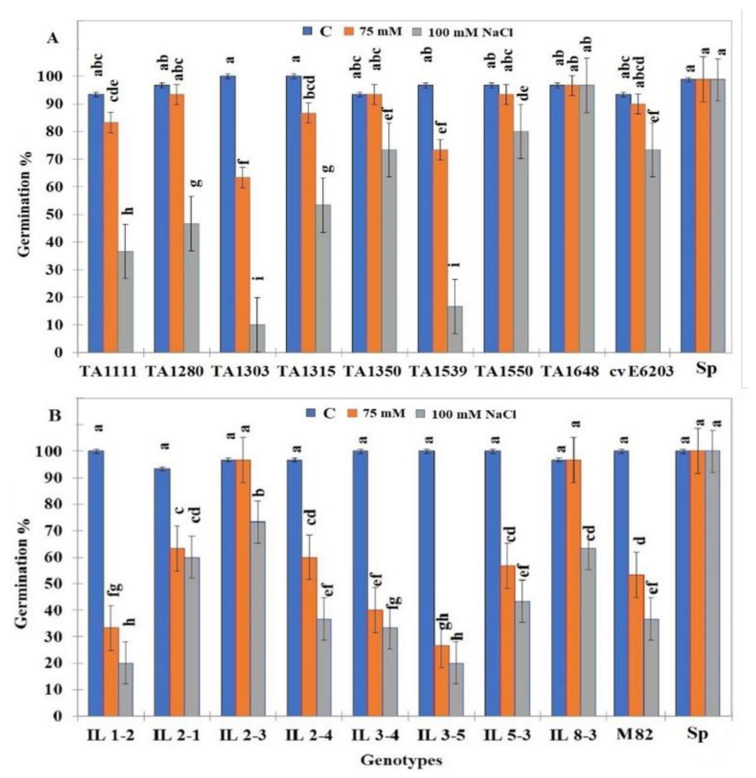
The percentage of germination of tomato ILs seeds under control (0 mM NaCl) and salt stress conditions (75 mM and 100 mM NaCl). (**A**) ILs from group I (*S. lycopersicum* cv-6203 × *S. habrochaites*). (**B**) ILs from group II (*S. lycopersicum* cv-M-82 × *S. pennellii*). Data are means of three replicates ± standard deviation; different letters on bars represent significant values according to Duncan’s test (*p* < 0.05).

Salt tolerance evaluation at the germination stage was performed at two concentrations of NaCl (75 and 100 mM). The germination of wild-type *S. pennellii* seeds was not affected even in the presence of 100 mM NaCl. However, seed germination of the two cultivated cultivars cv-E6203 and cv-M82 was negatively affected at a significant level by the presence of 100 mM NaCl. While at this concentration (100 mM NaCl), it was noticed that cv-E6203 was more tolerant than cv-M82 since their seeds germinated at 70% and 40%, respectively (Figure 1). Of the 16 ILs tested, only one from group I (TA1648) and three from group II (IL2-1, IL2-3, and IL8-3) showed a significantly higher percentage of germination either under 75 or 100 mM than their respective cultivated parent cv-E6203 and cv-M82 (Figure 1A,B). Most interestingly the percentage of germination of TA1648 was equal to control conditions at 75 or 100 mM NaCl as recorded for the salt-tolerant wild type *S. pennellii* (Figure 1). With regard to group II, the IL2-3 was the best tolerant line since it showed the significantly highest germination percentage at 75 and 100 mM NaCl.

### 2.2. Evaluation at Vegetative Stage under Hydroponics

#### 2.2.1. Morphological Traits

**Figure 2:**
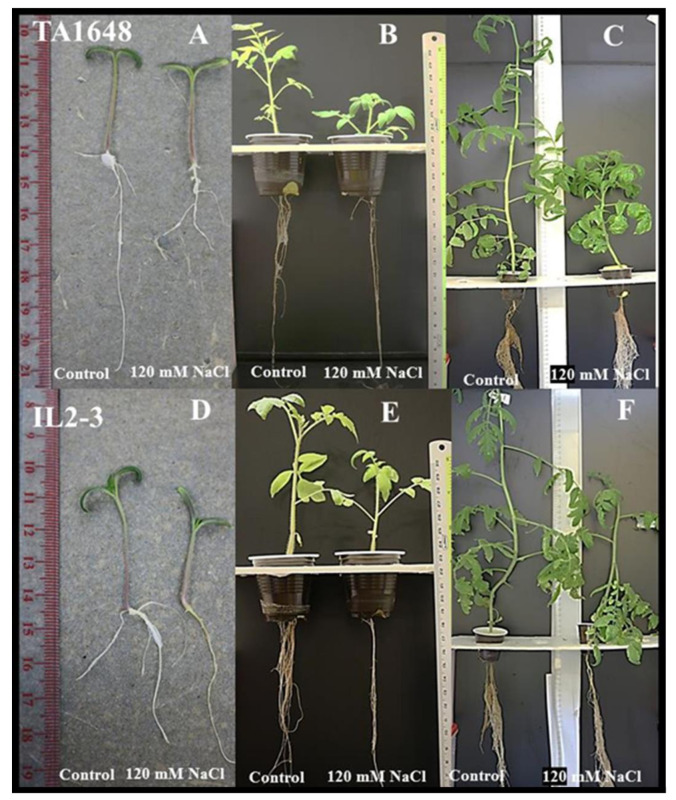
Salt tolerance evaluation in tomato genotypes under control and salt stress (120 mM NaCl). TA1648 and IL2-3 introgression lines at: Germination stage (**A**,**D**), seedling stage (**B**,**E**), and vegetative stage (**C**,**F**), respectively. The hydroponic growing system used for salt stress tolerance evaluation.

**Figure 3:**
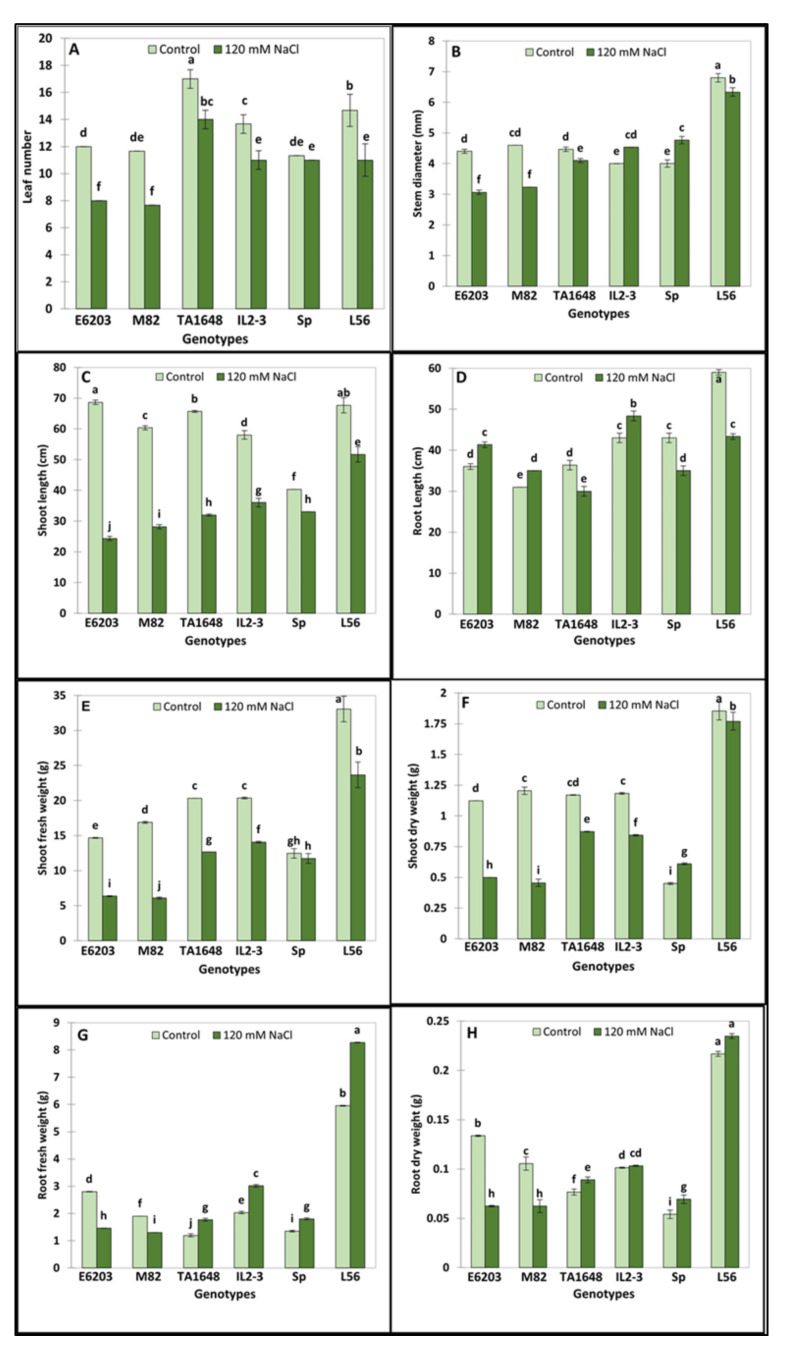
Salt tolerance evaluation under a hydroponic system of two ILs TA1648 and IL2-3 in comparison to two salt-sensitive cultivated tomato varieties “E6203, and M82” and two salt-tolerant reference genotypes, “LA0716” (wild type: SP) and “L56” (fixed variety). Two treatments control and 120 mM NaCl, were used. (**A**) Leaf number (LN), (**B**) stem diameter (SD), (**C**) shoot length (SL), (**D**) root length (RL), (**E**) shoot fresh weight (SFW), (**F**) shoot dry weight (SDW), (**G**) root fresh weight (RFW), and (**H**) root dry weight (RDW). Data are the means of three replicates ± standard deviation; different letters on bars represent the significant values according to Duncan’s test (*p* < 0.05).

Based on the results of salt tolerance at the germination stage, the two ILs with the highest percentage of germination (TA1648 and IL2-3) were selected for further evaluation at a vegetative stage under hydroponics at 120 mM NaCl during two months. In addition, their cultivated parents (cv-E6203 and cv-M82), the salt-tolerant check variety L56, and the wild-type SP were also used in this experiment (Figure 2). Variance analysis showed a significant treatment effect for all measured traits and genotypes by treatment interactions (Table S1). The values of the growth parameters leaf number (LN), shoot length (SL), shoot fresh weight (SFW), and shoot dry weight (SDW) significantly decreased following salt treatment for all tested lines except for LN, SFW, which did not show any significant variation in case of the wild SP genotype (Figure 3A,C,E,F). However, it is important to note that this reduction was significantly less important in tolerant lines (SP, L56, IL2-3, and TA1648) than in the cultivated sensitive lines (E6203, M82). The SD (stem diameter) increased by 20% and 13% for lines SP and its introgression line IL2-3 respectively, while the SD decreases for other tested lines under salt stress (Figure 3B). Under salt stress the root length (RL) increased significantly in IL2-3 and sensitive cultivated lines (E6203, M82) (Figure 3D). However, the RL decreases after salt stress for the salt-tolerant wild type SP and the cultivated variety L56 (Figure 3D). Moreover, the introgression line TA1648 showed the same comportment as a tolerant line for RL. From all the measured growth parameters, the root fresh weight (RFW) and root dry weight (RDW) represent the best traits that can be used to differentiate between tolerant and sensitive lines. Indeed, the RFW and RDW under salt stress significantly increased in tolerant lines (IL2-3, TA1648, L56, and SP) while they showed a significant reduction in sensitive cultivated lines (E6203, M82) (Figure 3G,H). 

#### 2.2.2. Chlorophyll Concentration

**Figure 4:**
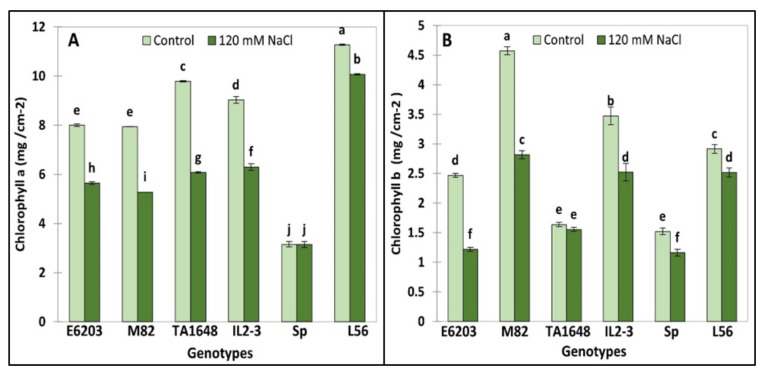
Chlorophyll concentration for two ILs (TA1648 and IL2-3), the cultivar parents (cv. E6203 and M82) and two salt-tolerant reference genotypes, “SP” (wild type) and “L56” (fixed variety) under control salt stress conditions (120 mM NaCl). (**A**) Chlorophyll a (Chl.a), (**B**) Chlorophyll b (Chl.b). Values are expressed as means ± SD of three plants per genotype. Different letters indicate statistically significant differences according to Duncan’s test (*p* < 0.05).

Chlorophyll a (Chl.a) and chlorophyll b (Chl.b) were analyzed in young leaves to evaluate salt tolerance of ILs. The Chl. a and Chl. b concentration significantly decreased under salt stress (120 mM NaCl) in all evaluated tomato ILs and salt-tolerant check variety L56 (Figure 4A,B). However, Chl.a in SP and Chl.b in TA1648 were not affected following salt stress (Figure 4). The lowest Chl.a reduction by about 10% was recorded in the tolerant cultivated tomato L56 followed by 30% in E6203 and IL2-3 (Figure 4A). However, for Chl.b the lowest decrease is about 5% in the IL TA1648 followed by 14% in the tolerant variety L56 (Figure 4B).

#### 2.2.3. Na^+^ and K^+^ Ions Concentration

**Figure 5:**
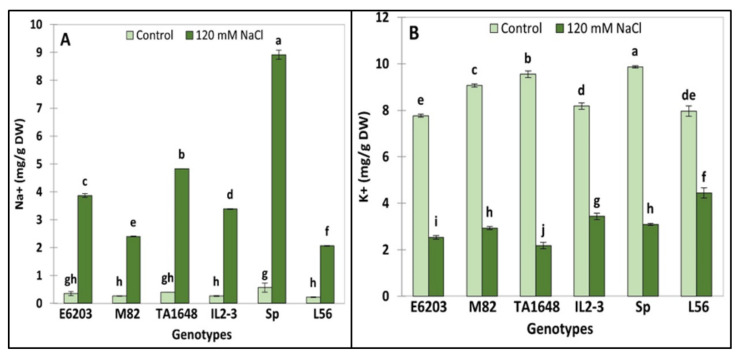
Ions (Na^+^ and K^+^) accumulation under control and salt stress (120 mM NaCl) in leaves of two ILs (TA1648 and IL2-3), the cultivar parents (cv. E6203 and M82), and two salt-tolerant reference genotypes, “SP” (wild type) and “L56” (fixed variety). (**A**) Sodium concentration (Na^+^), (**B**) Potassium concentration (K^+^). Values are expressed as means ± SD of three plants per genotype. Different letters indicate statistically significant differences according to Duncan’s test (*p* < 0.05).

Under salt treatment, the highest significant accumulation of Na^+^ was recorded in the wild type SP which represents 16 times more than in control leaves (Figure 5A). Under salt stress when compared to control conditions, the accumulation of Na^+^ ions was significantly different between the tested lines. Indeed, the highest accumulation was observed in the IL2-3 (Na^+^ accumulation 13-fold times more) followed by the TA1648 line (Na^+^ accumulation 12-fold times more). Finally, under salinity stress, the cultivated lines L56, M82, and E6203 accumulated Na^+^ between 9- to 11-fold times more than under the control conditions (Figure 5A). 

Under stress conditions, the highest K^+^ accumulation was observed in the tolerant check variety L56 (4.4 mg/g DW) followed by IL2-3 (3.4 mg/g DW), SP, M82, and finally by TA1648 and E6203 (Figure 5B). Based on the fact that Na^+^ affects K^+^ homeostasis involved in numerous metabolic processes, maintaining the equilibrated cytosolic Na^+^/K^+^ ratio is a key salinity tolerance mechanism. The highest Na^+^/K^+^ ratio was observed in the salt-tolerant wild-type genotype SP. (Na^+^/K^+^ = 2.9), while the lowest ratio was registered in the tolerant check variety L56 (Na^+^/K^+^ = 0.47). Finally, the ILs IL2-3 and TA1648 have Na^+^/K^+^ ratios of 1 and 2, respectively.

### 2.3. Principal Component Analysis for Growth Parameters

**Figure 6:**
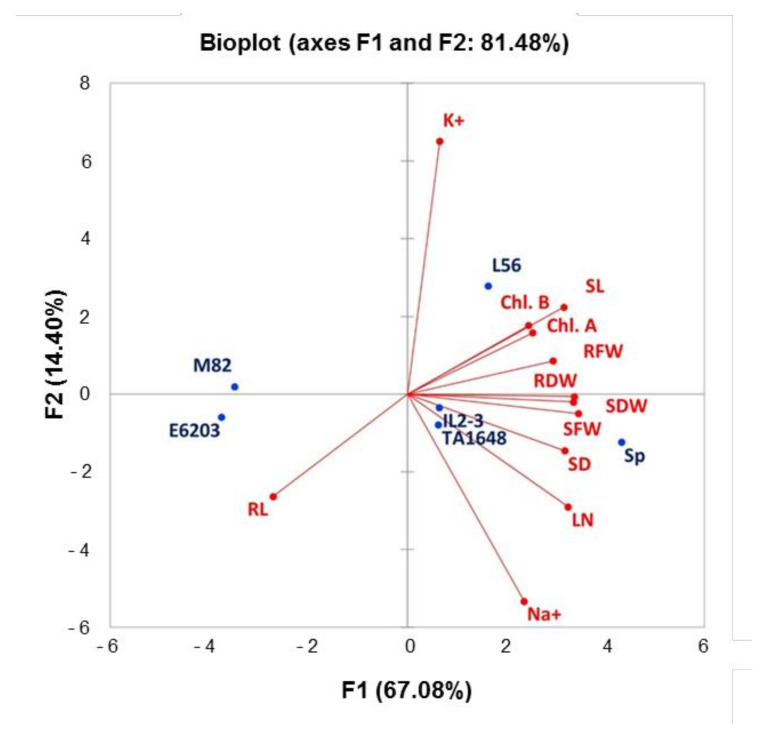
Principal components biplot (PC1 and PC2) for a salt tolerance index of 12 measured traits for six tomato genotypes grown under saline condition (120 mM). Leaf number (LN), shoot length (SL), stem diameter (SD), shoot fresh weight (SFW), shoot dry weight (SDW), root length (RL), root fresh weight (RFW), root dry weight (RDW), chlorophyll a (Chl.a), chlorophyll b (Chl.b), sodium leaf concentration (Na^+^), and potassium leaf concentration (K^+^).

Principal Component Analysis (PCA) was conducted for average values of salt tolerance indices (S/C) for all measured traits to determine the main growth parameters that could be estimated to evaluate and select the salt-tolerant tomato ILs (Figure 6). The first three principal components explained 92.63% of the phenotypic variation and covered all measured traits (Table S2). The first two principal components (PC1 and PC2) had Eigenvalue greater than 1.7 and explained 67.08% and 14.4% of the total variance, respectively. Ten growth traits with a score of >0.48 positively loaded in PC1 included measured traits, LN, SL, SFW, SDW, SD, RL RFW, RDW, Chla, and Chlb. While PC2 contained ions content parameters (Na^+^, and K^+^) with a high score >0.48 (Table S2). Indeed, SFW, SDW, and RDW under salt stress demonstrated the highest values in PCA1 which were significantly correlated in salt-tolerant genotypes (SP and L56). PCA1 had a positive correlation with all measured traits, except for one trait, RL which demonstrated a negative correlation with all other traits (Figure 6, Table S3). The Eigenvector’s distance and direction characterized the relationships between traits and genotypes. The scattering of tomato genotypes in the same direction helped group them with similar physiology traits associated with salt tolerance. PCA substantially demonstrated that RL has a stronger correlation with the salt-sensitive genotypes (E6203 and M82). Under salt stress 120 mM NaCl, the salt-tolerant genotype L56 moved towards a chlorophyll concentration, K^+^ concentration, SL, and RFW. The introgression lines, TA1648 and IL2-3 as well as wild SP (relative parent of IL2-3), were shifted toward the morphological traits, SFW, SDW, RDW, SD, LN, and Na^+^ concentration.

### 2.4. Agglomerative Hierarchical Clustering (AHC) Analysis for Salt Tolerance Indices

**Figure 7:**
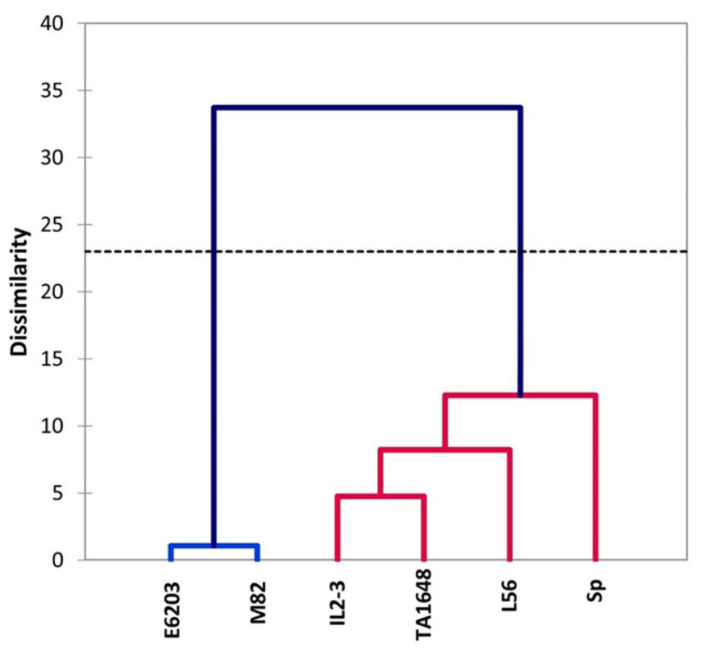
Hierarchical cluster analysis of two ILs (TA1648 and IL2-3), the cultivar parents (cv. E6203 and M82) and two salt-tolerant reference genotypes, “SP” (wild type) and “L56” (fixed variety) for salt tolerance indices based on physiological parameters analysis at the vegetative stage grown under control and salt stress condition (120 mM). The dendrography was drawn by Ward’s method in XLSTAT software.

The relationships between morpho-physiological changes under salt stress were used to cluster the salt tolerance of six tomato genotypes that included two salt-tolerant check genotypes. Hierarchical cluster analysis based on Euclidean distance classified the genotypes into two clusters (Figure 7). Cluster I included four genotypes classified for salt tolerance in this order SP > L56 > TA1648 > IL2-3. Sp has the lowest degradation level of morphological and physiological traits under salt stress when compared to non-salinized condition and this genotype was classified as highly salt-tolerant. In addition to three genotypes (L56, TA1648, and IL2-3) with minimal decrease in growth traits under saline condition. The last two genotypes (E6203 and M82) were located into cluster II which indicated the salt-sensitive genotype.

### 2.5. Expression Profile of Salt Responsive Genes in Tomato ILs

**Figure 8:**
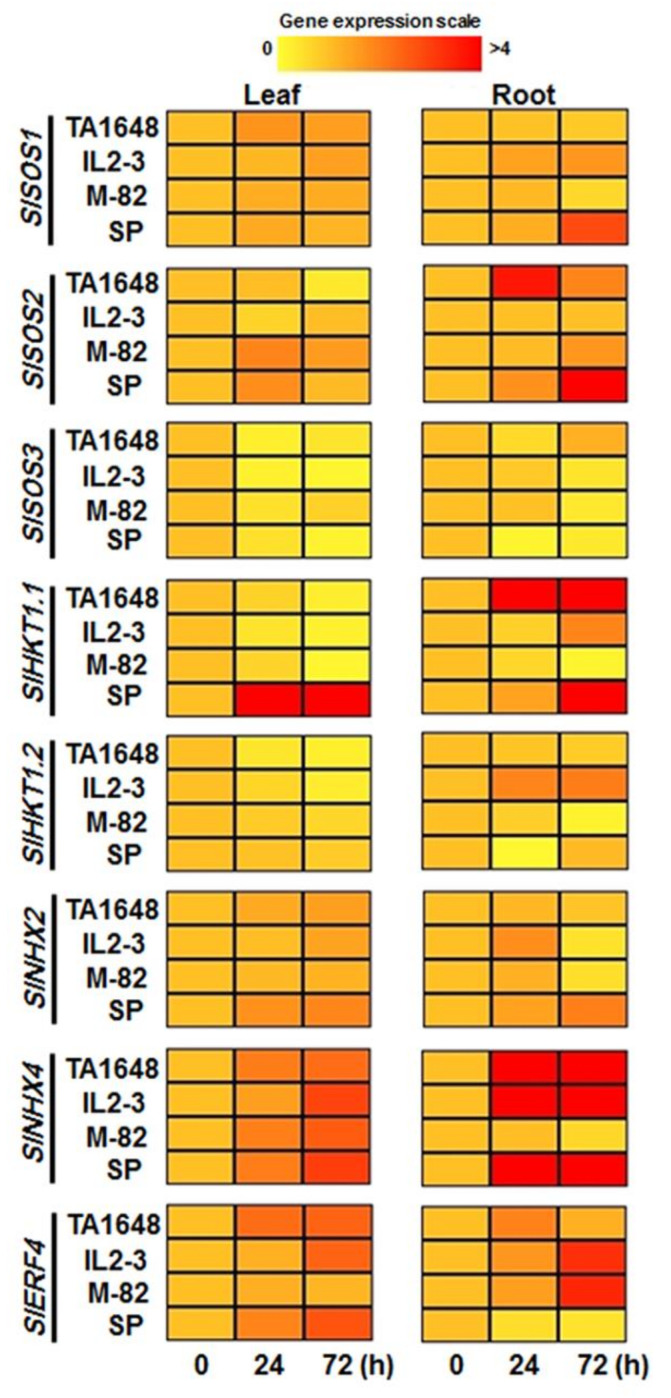
Heat map showing the expression level of eight salt stress-related genes throughout the leaf and root of tomato ILs and their relative parents in response to 120 mM NaCl treatment after 24 and 72 h. Data from qRT-PCR experiments were analyzed according to the 2^−∆∆Ct^ method. The housekeeping actin gene was used as an internal reference control to normalize the expression levels of the target genes. Values are mean ± SD. (n = 3) at *p* < 0.05.

The molecular response of two candidates salt-tolerant ILs, (TA1648 and IL2-3) were selected depending on the previous results of this study. In addition, *S. Pennellii* (SP) and M82 were examined. Expression profiles of eight salt stress-related genes *SlSOS1*, *SlSOS2*, *SlSOS3*, *SlHKT1.1*, *SlHKT1.2*, *SlNHX2*, *SlNHX4*, and *SlERF4* were investigated in the shoot and root samples in response to 120 mM NaCl after 24 and 72 h of treatment (Figure 8, Figure S1). The relative expression level of most of these genes increased with salt treatment in comparison with that of control plants, which means these genes are involved in plant response to salt stress. A comparison of the transcript accumulation level of the eight genes in shoot and root revealed that the salt overly sensitive (SOS), which is responsible for Na^+^ extrusion, particularly *SlSOS1*, was upregulated in leaves of all tested lines. In the roots it was upregulated in the tolerant lines of wild type SP and its relative IL2-3, while it was downregulated in the cultivated line M82 and its relative IL TA1648 (Figure 8). Compared to the control, SP relatively expressed *SlSOS1* and *SlSOS2* in root with 2.8-fold and 12.7-fold after 72 h of 120 mM NaCl treatment, respectively. For SlSOS3 it was downregulated in leaves and roots of all tested lines following salt treatment (Figure 8).

It is known that the expression of HKT can motivate the tolerance in the plant through Na+ exclusion from the shoot. After 24 and 72 hr of 120 mM NaCl, all tested tomato genotypes downregulated the expression level of *SlHKT1.1* and *SlHKT1.2* in leaves except for Sp, which upregulated the expression level of *SlHKT1.1* in shoot to 28 and 8.6-fold after 24 and 72 h, respectively. The expression pattern of *SlHKT1.1* in Sp, IL2-3, and TA1648 roots was significantly upregulated under salt stress, while it was downregulated in the root of the sensitive M82 line. The transcripts of *SlHKT1.1* gene were highest in the root of the TA1648 genotype with 15.4-fold after one day. However, for *SlHKT1.2*, it was upregulated only in the roots of the Il2-3 line. In the salt-sensitive M82, both *SlHKT1.1* and *SlHKT1.2* showed a downregulation in the root under salt stress (120 mM NaCl) (Figure 8). 

The vacuolar sequestration of sodium ions by the tonoplast Na^+^/H^+^ antiporter NHX is a significant mechanism defense in salt-tolerant plants to regulate the excess cytosolic Na^+^ under salinity condition. Interestingly, after salinity treatment of tomato seedlings, the transcripts level of *SlNHX2* and *SlNHX4* steadily increased in the leaves of both salt-tolerant and sensitive tomato genotypes over the time of exposure after 1 and 3 days (Figure 8). The *SlNHX4* relative expression in leaves was higher than that of *SlNHX2* after exposure to 120 mM NaCl of salt stress in all tested tomato genotypes. Importantly, under 120 mM NaCl, the salt-tolerant genotypes, TA1648, IL2-3, and, SP highly transcript *SlNHX4* in the root with 38-, 36-, and 7-fold and 49-, 22-, and 20-fold after 24h and 72 h, respectively. Contrary, *SlNHX4* was repressed in the root of the salt-sensitive tomato genotype M82. Finally, the expression pattern of the ethylene-responsive transcription factor (ERF4) which is important in salt stress response was analyzed. Under salinity treatment, the relative expression level of *SlERF4* was significantly increased in the leaves and roots of all tomato genotypes except the root of the SP genotype (Figure 8).

## 3. Discussion

Salt stress was defined as the presence of unsuitable salts, particularly NaCl in the environment surrounding the plant which negatively affects all plant growth stages. Salt stress causes a delay in seed germination and a decrease in the germination rate of tomato plants [26]. In this work, it was confirmed that the germination of the wild type tomato Sp seeds was not affected by the presence of 75 mM and 100 mM NaCl. However, for the sensitive cultivated tomato seeds of M82 and cvE6203, the germination rate was significantly decreased up to 38% and 72%, respectively. It is clear that the cultivated line cvE6203 is more salt tolerant than M82. These results are compatible with those obtained by Moles et al. [26]. With respect to all ILs from group I (*Solanum lycopersicum* cv-6203 × *Solanum habrochaites*), only one TA1648 showed better tolerance than its parent cvE6203 with an unaffected germination rate at 100mM NaCl when compared to control conditions. On the contrary, three lines from group II (*S. lycopersicum* M82 × *S. pennellii*) which are IL2-1, IL8-3, and IL2-3 and showing a germination rate of 60%, 62%, and 72% at 100 mM NaCl, respectively. These germination rates are almost twice that of the one of sensitive line M82, which is one of their parents. The decline in germination rate may be caused by osmotic stress (indirect negative effect of salinity), which limits water imbibition and determine metabolism activities [27,28]. This problem is followed by ions toxicity (Na^+^ and Cl^-^) that can cause enzymatic inhibition or changes in hormonal activities resulting in a decreased germination percentage [29,30]. 

The results indicated genotypic diversity in a seed germination rate and growth responses of tomato ILs seeds to salt stress. Indeed, the 100 mM NaCl was the best concentration to screen the ILs for salt tolerance when compared to their parents. In fact, between all tested ILs the following lines TA1648, IL2-1, IL2-3, and IL8-3 showed the best tolerance to salinity than the remaining ones. These results are in agreement with those reported by Uozumi et al., [31] who demonstrated that IL8-3 had a higher germination rate in comparison with cv M-82 under salt stress. The variance in salt tolerance between tomato ILs is generally attributed to the segments of genetic backup from their wild parent, *S. Pennellii* [9] or *S. habrochaites* [32,33]. Introgression lines consist of a genomic proportion of a wild tomato parent replaced by homologous regions in the background of cultivated tomato species but without most of the unfavorable traits of the wild species [34]. The enhancement of salt tolerance in the selected ILs can be explained by the presence of salt stress related genes derived from the wild type parents *S. pennellii* or *S. habrochaites* genome. 

It is of high priority to identify salt-tolerant ILs suitable to be selected as rootstocks for tomato cultivars, which will improve the growth and fruit quality under salt stress conditions. Most commercial tomato cultivars are sensitive to moderately salt-tolerant [35]. However, *S. Pennellii* has been recognized as a halophytes plant [36]. In this study, six tomato genotypes that included two ILs with a high germination percentage were selected for evaluation at a vegetative stage evaluation under (120 mM NaCl). Salt stress negatively impacted LN, SL, SFW, SDW, RFW, and RDW, except for SP, the reduction in SFW and LN traits was significant in all tested genotypes. The salt-tolerant genotype (SP) had higher SDW and RDW (36% and 28%, respectively) than the control plants. Genotypes, (TA1648, L56, and IL2-3) had a higher RDW (16%, 10%, and 2%, respectively) than the control plants. In addition, the reduction in SFW and SDW was higher than that of RFW and RDW. This finding agrees with Foolad [37] who mentioned that salinity reduced shoot growth more than root growth. The increase in dry matter of salt-tolerant tomato indicates its ability to accumulate organic molecules such as osmoprotectants, polysaccharides, and amino acids inside the leaf and root under salinity conditions [14]. Pailles et al., [38] reported that salt-tolerant and wild tomato species, *S. cheesmaniae* and *S. galapagense* genotypes were able to maintain growth (based on dry mass) during the salt stress condition better than the cultivated tomato. Maggio et al., [39] reported that root dry matter was greater by increasing the salinity (especially after 9 dS m^−1^) in tomatoes. Salinity is a major factor limiting plant development at different stages, which negatively decreases the yield productivity [40]. Saline irrigation water contains sufficient amounts of harmful soluble salts and can suppress plant growth through osmotic potential effect and trigger antagonism with nutrient and causes ion toxicity [41]. Salt stress causes several adverse effects on plant physiological characteristics such as water balance, growth rate, ions uptake, and photosynthetic system, which results in yield reduction. Morphological traits including leaf number, stem diameter, and plant height are also affected [42].

The results of this study indicated a decrease of chl.a and chl.b concentration in all genotypes. Reducing the chlorophyll pigments as a response to abiotic stress causes a reduction in the net photosynthesis and thus in energy, which is very important for metabolism and growth. Abiotic stress also causes serious adverse effects on other photosynthesis mechanisms including stomatal dysfunction and limiting CO2 supply, turgor loss, and photosynthesis enzyme deactivation [43]. K^+^ is a critical factor in stomatal aperture regulation, which is needed to permit the plant to uptake sufficient CO2 for photosynthesis and control transpiration to manage water uptake and loss under salt stress conditions [44]. Several authors reported that abiotic stress (salinity, drought, and heat stress) negatively affects photosynthesis and plant growth [43,45,46,47].

Sodium accumulation is induced by salt stress in nearly all evaluated plants. However, the quantity of Na^+^ in SP was significantly 2.6-fold higher than the values of its relative IL2-3. The K^+^ uptake of IL2-3 was significantly greater than that of SP under salinity conditions. This finding is supported by Frary et al., [48] who reported that IL2-1 and IL11-3 exhibited decreased Na^+^ content under salinity condition, unlike *S. pennellii*. These findings may indicate that these genotypes inherited a genome segment from chromosomes 2 and 11 of its parent *S. pennellii* containing mutations for ion uptake/transport causing sodium uptake deficiency. IL2-3 exhibited a higher salt tolerance index with higher K^+^ content than the recurrent parent and lower Na^+^ accumulation under both control and salt stress [49].

To identify the useful and most correlated growth traits contributing to salt tolerance and to classify the evaluated ILs for tomato improvement, principal component analysis (PCA) was carried out [50]. There was an evident distribution between introgression lines in the PCA for the salt tolerance index of growth traits, with greater participation of shoot and root matter in salt-tolerant genotypes (Table S2). The most remarkable variance was in RFW and RDW, which were the highest in SP, L56, IL2-3, and TA1648 with salt treatment (Figure 3). As expected, hierarchical clusters collect these ILs together in the same group including the salt-tolerant wild type, *S. Pennellii*, and salt-tolerant cultivar, L56. In other reports, treated tomatoes with salinity stress, plant height, and leaf number were positively correlated in PC1 and root length was positively correlated in PC2 to salt tolerance [51]. In this study, the salt-tolerant genotypes were specified with increased fresh and dry root weight under saline conditions in comparison to control plants. A negative association was observed between Na^+^ and chlorophyll (Chl.a and Chl.b), which is confirmed by other studies [52,53,54]. This study indicated that an increase in Na^+^ concentration leads to a decrease in K^+^ in tomato shoot in salt-treated plants (Figure 5). Similarly, it has been reported that under high salt stress, high Na^+^ content inside plant causes inhibition in K+ uptake [55,56].

Under salt stress, the plants face the problem of a high level of Na^+^ accumulation in the cell cytoplasm, which can cause dysfunction in many important cellular metabolites [21]. Many salt responsive genes are expressed as cell signaling [57], transcription factors [58,59], and transporter proteins [18,60], which can play an essential role in maintaining these toxic ions away or inside vacuoles. The first functional analysis for salt overly sensitive (SOS) genes including SOS1, SOS2, and SOS3 is to improve its role in ion homeostasis under salt stress was reported by Zhu et al., [61]. Many studies were conducted to decipher the main function for each gene in SOS pathway and salt tolerance, SOS1 was identified in bacteria, fungi, and plants as a plasma membrane Na^+^⁄H+ antiporter and act a key role in sodium efflux from root cells, predominantly root tip to shoot [62,63,64]. The SOS pathway start after receiving the salt stress signal at the early response by the Ca2^+^ binding protein, SOS3 which activates a serine/threonine-protein kinase, SOS2. The SOS1 was reported to be regulated by SOS2 and an increasing expression level in response to salinity. The active SOS2 protein phosphorylates Na^+^/H^+^ antiporter SOS1 on a plasma membrane to dispose of higher a Na^+^ [55,65]. These reports support the finding of this study. It is reported that the transcript level of SOS genes in root was higher than shoot in all genotypes. Salt-tolerant (*S. Pennellii*) showed *SlSOS2* and *SlSOS1* upregulation in root tissue by 12.7- and 2.8-fold, respectively after 72 h of 120 mM NaCl treatment. The salt-tolerant genotypes SP, TA1648, and IL2-3 increased the gene expression of *SlSOS1* in the root while the salt-sensitive M82 represented a low expression level of *SlSOS1* in root under excess Na+ ions in the growing condition. These results suggest that *SlSOS1* is involved in Na^+^ exclusion from the root and improved salt stress tolerance in these genotypes. Under salt stress, the upregulation of *SlSOS1* was observed in shoot of all salt-tolerant and salt-sensitive genotypes. These results support the concept that SOS1 is also localized in the stem and considered to mediate Na^+^ loading in xylem of both glycophytes and halophytes, particularly under salt stress [66,67].

Once the Na^+^ ions up take from the root, sodium is transported through xylem vessels to shoot and translocated between plant cells through the high-affinity K^+^ transporter HKT [68,69,70]. Phylogenetic and functional analyses manifested that HKT transporters consist of two subgroups: HKT1 identified as the Na^+^ uniport, and HKT2 identified as the Na^+^/K^+^ symport [71,72]. In this study, two tomato class I HKT genes *SlHKT1.1* and *SlHKT1.2* were analyzed, the salt-tolerant genotypes, SP, and tested ILs, TA1648 and IL2-3 had upregulation in *SlHKT1.1* in the root while they expressed at a lower level of *SlHKT1.2* (Figure 8). After subjecting the tested tomatoes to 120 mM NaCl treatment, TA1648 had a higher expression of *SlHKT1.1* in the root by 15 fold compared to the control plants. Furthermore, IL2-3 showed upregulation for both *SlHKT1.1* and *SlHKT1.2* in the root by about 2 fold, interestingly IL2-3 and TA1648 accumulate Na^+^ in the shoot lower than the halophyte genotype SP, which represented downregulation for *SlHKT1.2* and upregulation of the *SlHKT1.1* transcript in the root. HKT genes play an important role in avoiding the translocation of sodium from roots to shoot via xylem vessels [73]. Almeida et al., [74] reported that the higher transcript of *SlHKT1.2* resulted in reduced sodium accumulation in the *S. lycopersicum* shoot compared to wild-type *S. pennellii* (SP). It has been reported that the expression of *SOS1* and *HKT1* are upregulated under salinity conditions and mediate opposite fluxes of sodium ions across the plasma membrane [67,75].

Under salt stress conditions, the plant with the ability to maintain electrostatic balance via controlling ions (particularly Na^+^) inside the cell at optimum level can protect the cellular enzymatic activities and maintain salt tolerance [13,71]. NHX, cation/proton antiporter, or sodium/hydrogen exchanger is the gene family that is responsible for reducing the excess Na^+^ in cytoplasm by exchanging with H^+^ via vacuoles antiporters (NHXs) at the tonoplast and providing osmotic pressure inside the cells [13,76]. In this study, the relative expression level of *SlNHX2* was found higher in the leaf compared to root tissues, while the transcript of *SlNHX4* in the root was maximized to a great level of upregulation in response to salt stress in genotypes, TA1648, IL2-3, and SP by 48-, 21.7-, and 20.4-fold at 72 h while the expression level of the leaf peaked for approximately 3-folds. The *S. Pennellii* plants retained *SlNHX2* and *SlNHX4* transcripts with upregulation in both shoot and root in positive relation with time of exposure to 120 mM NaCl. This suggests that *S. pennellii* plants imprison the excess sodium into vacuoles, and this finding may explain the high level of Na^+^ concentration in the shoot as compared to other tested tomato genotypes. The vacuolar sequestration of sodium ions remains the cellular component undamaged and active. This idea might decipher why the chl.a concentration was not significantly decreased under salinity in *S.pennellii*. Albaladejo et al. [77] reported an increase in *NHX3* and *NHX4* transcript levels in wild tomato *S. pennellii* after 7 days of 100 mM NaCl treatment compared to *S. lycopersicum*. The enhanced transcription level of *SpNHX3* and *SpNHX4* in *S. pennellii* leaves might be responsible for enabling plants to sequester excess sodium into vacuoles under a salinity environment. The transgenic tomato with constitutive overexpression of *SlSOS2* revealed salt tolerance with association of high Na^+^ concentration in the shoot and with influencing the upregulation of *SlSOS1*, *SlNHX2*, and *SlNHX4* [78]. 

ERFs belong to a large family of plant transcription factors that are exclusively found in plants. ERF-TFs activate the expression of abiotic stress-responsive genes [79]. The upregulation of *SlERF4* in salt-tolerant tomato, SP, IL2-3, and TA1648 was higher than the transcript in cultivated tomato M-82 under salt stress. The expressions of soybean transcription factor, *GmERF4* were upregulated under salt stress. Transgenic tobacco with constitutive expressing *GmERF4* showed higher tolerance to salt stress compared with wild-type plants [80]. The results indicated that the upregulation of SlERF4 accompanied with the downregulation of *SlSOS3*, where the regulation between SOS pathway and ERF signaling in salt stress responses requires further studies [81].

## 4. Materials and Methods

### 4.1. Plant Material and Growth Conditions

Two groups of tomato introgression lines (ILs) and their cultivated (cv-6203, cv-M82) parents in addition to the wild-type *S. pennellii* (SP) were obtained from the Genetics Resource Centre (TGRC, University of California, Davis, Department of Plant Sciences, USA). Group-I and group-II represent the result of the following crosses respectively: *S. lycopersicum* (cv-6203) × *Solanum habrochaites* and *S. lycopersicum* (cv-M82) × *S. pennellii*. Group I is represented by the following lines: TA1111, TA1280, TA1539, TA1303, TA1315, TA1550, TA1350, and TA1648, which contain a homozygous chromosome segment from *S. habrochaites* (LA1777) in the background genome of *S. lycopersicum* (cv-E-6203: LA4024) [9]. In contrast, group II is represented by the following lines: IL1-2, IL2-1, IL2-3, IL2-4, IL3-4, IL3-5, IL5-3, and IL8-3, which contain a homozygous chromosome segment from the genome of *S. pennellii* (LA0716) in the background genome of *S. lycopersicum* (cv-M-82: LA3475) background [32,33]. Finally, salt-tolerant check variety L56 was also used. This genotype L56 was produced using self-pollination for six generations from the commercial cultivar Strain-B and selection for salt tolerance (tomato breeding program at the Vegetable Improvement Unit, College of Food and Agricultural Sciences, King Saud University) [82,83]. 

All ILs were grown for one season (September 2017 to March 2018) for seed multiplication in a greenhouse located at King Saud University (KSU) (Riyadh, KSA; 24.722° N 46.627° E). Standard agronomic practices were carried out during the growing season for all plots. The fruit was harvested at a mature red stage. New ILs seeds were used in salt tolerance evaluation. 

### 4.2. Evaluation at Germination Stage

Seeds from all genotypes were sterilized by immersion in a solution of 50% commercial bleach (5% sodium hypochlorite) for 15 min and then washed three times with sterilized deionized water for 15 min each and air-dried for 1 h. The sterilized seeds were sown in Petri dishes (90 mm diameter) containing a solid half-strength MS medium (0.5MS) [84]. The evaluation of salt tolerance at the germination stage was carried out in a Petri dish experiment as a complete randomized design. For this purpose, three salt treatments (0, 75, 100 mM NaCl), eight ILs from each group (I and II), the cultivated parents (cv-E6203 (LA4024), cv-M82 (LA3475)), and finally the salt-tolerant wild type *S. pennellii* (Sp) were used. Ten tomato seeds were placed in each Petri dish with three replications for each treatment and each line and incubated in a growth chamber at 24 °C and in a 16/8 hours’ light/dark photoperiod using cool white light (200 µmol m^−2^ s^−1^). The percentage of germination was calculated based on seedlings with developed cotyledons divided by the total number of germinated seeds. 

### 4.3. Evaluation at Vegetative Stage under Hydroponics

Based on seed germination evaluation, six genotypes were selected for vegetative stage evaluation under hydroponics: Two ILs (TA1648 from group I, IL2-3 from group II), two cultivated varieties (cv-E6203 and cv-M82), the salt-tolerant wild type *S. pennellii* (Sp) (LA0716), and the salt-tolerant check genotype L56. 

The experiment was performed in a controlled growth chamber. The surface-sterilized seeds were germinated in boxes (1 L) containing sterilized tissue paper under control and salt stress conditions (0 and 120 mM NaCl). Healthy tomato seedlings were transferred into sponge cubes inside small plastic cups. Two-week-old plantlets were transplanted into a hydroponic growing system containing half strength of nutrient solution (0.5NS) as previously described by Tarroum et al., [85]. In this experiment, six tomato genotypes (TA1648 from group I, IL2-3 from group II, two cultivated varieties cv-E6203 and cv-M82, the salt-tolerant wild type *S. pennellii* (Sp) (LA0716), and the salt-tolerant check variety L56) were cultivated in 0.5NS with or without 120 mM NaCl using a hydroponic system. In each condition, six plants were used as replications. The concentration of nutrient solution and salinity remained constant during the experiment. The plants were grown under growth chamber environmental conditions at 25/22 °C during the day/night with 16/8 h as photoperiod using cool white light (200 µmol m^−2^ s^−1^), and 70% relative humidity (RH). Finally, after two months of growing plants were harvested, and the morph-physiological traits were determined. 

#### 4.3.1. Morphological Traits

At the end of the experiment, three whole plants from each treatment were selected. Plants were divided into shoots and roots and the following parameters were determined for each plant: Plant height (PH), Root length (RL), leaf number (LN), stem diameter (SD), shoot fresh weight (SFW) and root fresh weight (RFW), and finally dry weights of the shoots (SDW) and roots (RDW). 

#### 4.3.2. Chlorophyll Concentration

Leaf discs from top developed leaves were cut and incubated in 80% acetone (v/v) at 4 °C for 72 h. The chlorophyll concentration in the solution was measured using a Spectrophotometer (Ultraspec 2100 pro, Amersham Bioscience, Cambridge, UK) by reading the absorbance (A) at 645 and 663 nm. Three replicates of individual samples were analyzed. Chlorophyll a (Chl.a) and chlorophyll b (Chl.b) concentration (mg.cm^−2^) were calculated according to Arnon [86] using the following equations:

Chl a (µg.cm^−2^) = [(12.7 × A663) − (2.6 × A645)] × ml of Acetone 80% / leaf area (cm^2^),

Chl b (µg.cm^−2^) = [(22.9 × A645) − (4.68 × A663)] × ml of Acetone 80% / leaf area (cm^2^).

#### 4.3.3. Ions Concentration

The quantity of dried leaves 0.2 g was mineralized in 2 mL 0.5% HNO3 as previously described by Ben Romdhane et al., [87]. The samples were centrifuged at 15,000 rpm for 30 min followed by a dilution with deionized water of 1/200 or 1/400 for control or salt stress, respectively. The concentration of Na^+^ and K^+^ was measured using a flame spectrophotometer (BWB XP Flame Photometer, BWB Technologies, England). Ion concentrations were calculated according to the standard curve and the results were expressed in mg/g of dry matter.

#### 4.3.4. Gene Expression

Surface sterilized seeds of TA1648, IL 2-3, M82, and SP were germinated and transferred to the hydroponic growing system under control conditions as previously described. Two weeks later, the control samples from leaves and root tissue were taken as control. Plants were treated with salt stress (120 mM NaCl). Leaves and roots from stressed plants were sampled at 24 and 48 h after treatment and immediately frozen in liquid nitrogen and stored at −80 °C for RNA extraction.

Total RNA was extracted using an SV Total RNA isolation kit (Promega, Madison, USA) according to the manufacturer’s instructions, and finally, the residual genomic DNA was removed by DNaseI enzyme. First-strand cDNA was synthesized with the ProtoScript II Kit (NEB, Hitchin, UK) using 1 µg of total RNA from two different biological replicates for each treatment according to the supplier’s standard protocol. cDNA was diluted (1:10) with nuclease-free water (Promega, Madison, USA) and used as a template for qRT-PCR reactions. Each reaction was performed in 15 µL containing: 7.5-µL Luna Universal qPCR Master Mix (NEB, Hitchin, UK), 2-µL cDNA, 2-µL Primer pairs 10 µM, and 3.5 µL of water. The experiments were done in three technical replicates. The qPCR reactions were carried out in the Light Cycler 480 instrument (Roche, Mannheim, Germany) using the following protocol: 95 °C for 3 min, followed by 40 cycles of 95 °C for 20 s, 55 °C for 20 s, and 72 °C for 30 s. A melting curve at 60–95 °C was routinely performed to verify primer specificity. As an internal reference control, the housekeeping actin gene was used to normalize the expression levels of the target genes. The primers of the eight tested salt-responsive genes and the internal reference gene used in qPCR reactions are listed in Table S4 [88,89,90]. The relative quantification of gene expression was calculated using the 2^−ΔΔCT^ method [91].

### 4.4. Statistical Analyses

A two-way analysis of variance (ANOVA) was performed to determine the effect of two salt stress levels on the percentage of germination rate of 16 tomato introgression lines, one wild-type parent, and two cultivated parents. The significant difference between the means of three replicates for all variables of interest was determined. The relative values of growth parameters including morphological and physiological traits for selected six genotypes which were evaluated during the vegetative stage into a hydroponic growing system under 120-mM NaCl were subjected to one-way analysis of variance (ANOVA). Duncan’s multiple comparison tests at *p* < 0.05 were used. Principal component analysis (PCA), and hierarchical clustering were performed using XLSTAT statistical software. Different letters were used to indicate means that differ significantly at *p* ≤ 0.05.

## 5. Conclusions

The development and identification of tomato salt-tolerant genotypes are instant requests to overcome the challenges of tomato production in arid regions that face complex limiting factors such as scarcity of freshwater, salinity, and heat stress. In this study, we evaluated 16 tomato introgression lines from two different groups. Our results concluded that the impact of salt stress on tomato ILs depends on the level of salt stress and genetic background inherited from the salt-tolerant wild-type parent that provides the mechanisms of salt tolerance. A negative affect was generally observed in plant growth traits under salt stress. Based on a statistical analysis of phenotypical and physiological parameters at the vegetative stage, tomato ILs TA1648 and IL2-3 compared with two reference salt-tolerant genotypes with one wild type (SP) and another cultivar (L56), appear to have an interesting salt-tolerance mechanism. These two lines TA1648 and IL2-3 could be used as salt-tolerant rootstock for grafting sensitive cultivated varieties or integrated in salt-tolerant breeding programs.

## Data Availability

Data is contained within the article or supplementary material.

## Supplementary Materials

The following are available online at https://www.mdpi.com/article/10.3390/plants10122594/s1, Table S1. Analysis of variance (ANOVA) (mean square) for morpho-physiological traits in six tomato genotypes at the vegetative stage under control (C) and salt stress (S) with 120-mM NaCl conditions. Table S2. PCA of six tomato genotypes, eigenvalues, proportion, and cumulative variance for the first four principal components for salt tolerance indices (S/C) from 12 growth traits. Table S3. Phenotypic correlation coefficients (r) values of the different pairs of estimated growth parameters of six tomato under non-saline and saline irrigation (120 mM) into hydroponic growing system. Table S4. Primers for gene expression analysis in tomato used in qPCR reactions. Figure S1. Expression profiles of eight salt stress-related genes in tomato ILs and its relative parents in response to 120-mM NaCl after 24 and 72 h of treatment. Data from qRT-PCR experiments were analyzed according to the 2^−∆∆Ct^ method. The housekeeping actin gene was used as an internal reference control to normalize the expression levels of the target genes. Vertical bars indicate standard deviation calculated from three replicates. Values are mean ± SD. (n = 3) at *p* < 0.05.
