# Supplementary material for: Analysis of Salinity Tolerance in Tomato Introgression Lines Based on Morpho-Physiological and Molecular Traits
Source: Plants (Basel). 2021 Nov 26;10(12):2594. doi: 10.3390/plants10122594 (PMC8704676; doi:10.3390/plants10122594)
Supplement: Supplementary file 1 [file plants-10-02594-s001.zip › plants-1440487-supplementary.pdf]

Table S1. Analysis of variance (ANOVA) (mean square) for morpho-physiological traits in six tomato genotypes at the vegetative stage under control (C) and salt stress (S) with 120mM NaCl conditions.

SOV	df	LN	SD (mm)	SL (cm)	RL (cm)	SFW (g)	SDW (g)	RFW (g)	RDW (g)	Chl.a (mg / cm^2)	Chl.b (mg /cm^2)	Na+ (mg/g DW)	K+ (mg/g DW)
Genotypes (G)	5	27.78**	6.37**	333.91**	306.24**	264.31**	1.13**	28.53**	0.02**	35.62**	5.04**	10.31**	1.21**
Treatment (T)	1	78.03**	1.25**	6045.06**	58.78**	466.56**	0.93**	1.42**	0.00	39.92**	5.74**	136.77**	285.44**
G X T	5	2.89**	1.21**	269.20**	114.98**	18.58**	0.17**	2.43**	0.00	2.57**	0.60**	8.46**	2.99**
Error	24												

leaf number (LN), stem diameter (SD), shoot length (SL), root length (RL), shoot fresh weight (SFW), shoot dry weight (SDW), root fresh weight (RFW), root dry weight (RDW), chlorophyll a (Chl_a), chlorophyll $_b$ (Chl_b), Sodium leaves content (Na+), potassium leaves content (K^+). Df, degree of freedom. ** significant at 1% level of significance.

Table S2. PCA of six tomato genotypes, Eigenvalues, proportion, and cumulative variance for the first four Principal components for salt tolerance indices (S/C) of twelve growth traits.

	**PCA1**	**PCA2**	**PCA3**	**PCA4**
Eigenvalue	8.050	1.728	1.457	0.689
Variability (%)	67.080	14.402	12.144	5.739
Cumulative %	67.080	81.482	93.626	99.364
LN	**0.853**	0.146	0.000	0.001
SL	**0.811**	0.087	0.065	0.003
SD	**0.817**	0.037	0.005	0.140
SFW	**0.964**	0.004	0.021	0.002
SDW	**0.910**	0.001	0.050	0.039
RL	**0.591**	0.119	0.065	0.217
RFW	**0.700**	0.013	0.196	0.091
RDW	**0.921**	0.000	0.078	0.001
Chl.a	**0.516**	0.043	0.374	0.067
Chl.b	**0.483**	0.054	0.456	0.006
Na+	0.449	**0.491**	0.045	0.005
K+	0.035	**0.734**	0.102	0.118

Values ≥ 0.48 are presented in bold-face and indicate traits important for PC. leaves number (LN), shoot length (SL), stem diameter (SD), shoot fresh weight (SFW), shoot dry weight (SDW), root length (RL), root fresh weight (RFW), root dry weight (RDW), chlorophyll a (Chl_a), chlorophyll $_b$ (Chl_b), Sodium leaves content (Na+), potassium leaves content (K^+).

Table S3. Phenotypic correlation coefficients (r) values of the different pairs of estimated growth parameters of six tomato under non-saline and saline irrigation (120 mM) into hydroponic growing system.

Variables	**LN**	**SL**	**SD**	**SFW**	**SDW**	**RL**	**RFW**	**RDW**	**Chl. A**	**Chl. B**	**Na+**	**K+**
LN	**1**											
SL	0.725	**1**										
SD	**0.899**	0.798	**1**									
SFW	**0.931**	**0.885**	**0.925**	**1**								
SDW	**0.900**	**0.899**	0.810	**0.962**	**1**							
RL	-0.586	-0.688	-0.434	-0.684	-0.758	**1**						
RFW	0.715	0.685	**0.814**	0.764	0.636	-0.656	**1**					
RDW	**0.886**	0.798	**0.859**	**0.901**	**0.848**	-0.790	**0.933**	**1**				
Chl. A	0.599	**0.853**	0.558	0.768	**0.868**	-0.587	0.275	0.511	**1**			
Chl. B	0.548	0.523	0.505	0.564	0.521	**-0.822**	**0.882**	**0.852**	0.156	**1**		
Na+	**0.885**	0.436	0.782	0.748	0.691	-0.194	0.411	0.589	0.446	0.152	**1**	
K+	-0.163	0.499	0.153	0.197	0.158	-0.208	0.216	0.089	0.416	0.083	-0.372	**1**

Values in bold are different from 0 with a significance level alpha=0.05. leaves number (LN), shoot length (SL), stem diameter (SD), shoot fresh weight (SFW), shoot dry weight (SDW), root length (RL), root fresh weight (RFW), root dry weight (RDW), chlorophyll a (Chl_a), chlorophyll $_b$ (Chl_b), Sodium leaves content (Na+), potassium leaves content (K^+).

Table S4. Primers for gene expression analysis in tomato used in qPCR reactions.

No	Primers	Sequence 5′-3′	Reference
**1**	SlSOS1-F	TCGAGTGATGATTCTGGTGG′	Huertas et al. [78]
**2**	SlSOS1-R	ATCACAGTGTGGAAAGGCT′	
**3**	SlSOS2-F	CTGCTTAGGACAAGGACTCG′	Huertas et al. [78]
**4**	SlSOS2-R	GGTATAGTGTGTGTAACTGC3′	
**5**	SlSOS3-F	GCAACGGAGTGATTGGATTTG	Qi et al., [23]
**6**	SlSOS3-R	CCATCTCTTTCAGCTCTTCTCTC	
**7**	LeNHX2-F	CCTTTGAGGGGAACAATGG′	Huertas et al. [78]
**8**	LeNHX2-R	CATCTTCATCTTCGTCTCC′	
**9**	LeNHX4-F	TGGTGGGCAGGTTTGATGAGAG	Huertas et al. [78]
**10**	LeNHX4-R	TGTGGTGGCAGCAGGAGACTTA	
**11**	SlHKT1.1-F	TCTAGCCCAAGAAACTCAAAT	Asins et al. [89]
**12**	SlHKT1.1-R	CTAATGTTACAACTCCAAGGAATT	
**13**	SlHKT1.2-F	TGAGCTAGGGAATGTAATAAACG	Asins et al. [89]
**14**	SlHKT1.2-R	AGAGAGAAACTAACGATGAACC	
**15**	SlERF4-F	ACAGATCCATTTACGCATAAGAGAGTCC	Liu et al. [90]
**16**	SlERF4-R	GCTGAATATGATCGCAATTCTTCTTCAG	
**17**	SlActin-F	TTGCTGACCGTATGAGCAAG	Qi et al., [23]
**18**	SlActin-R	GGACAATGGATGGACCAGAC	

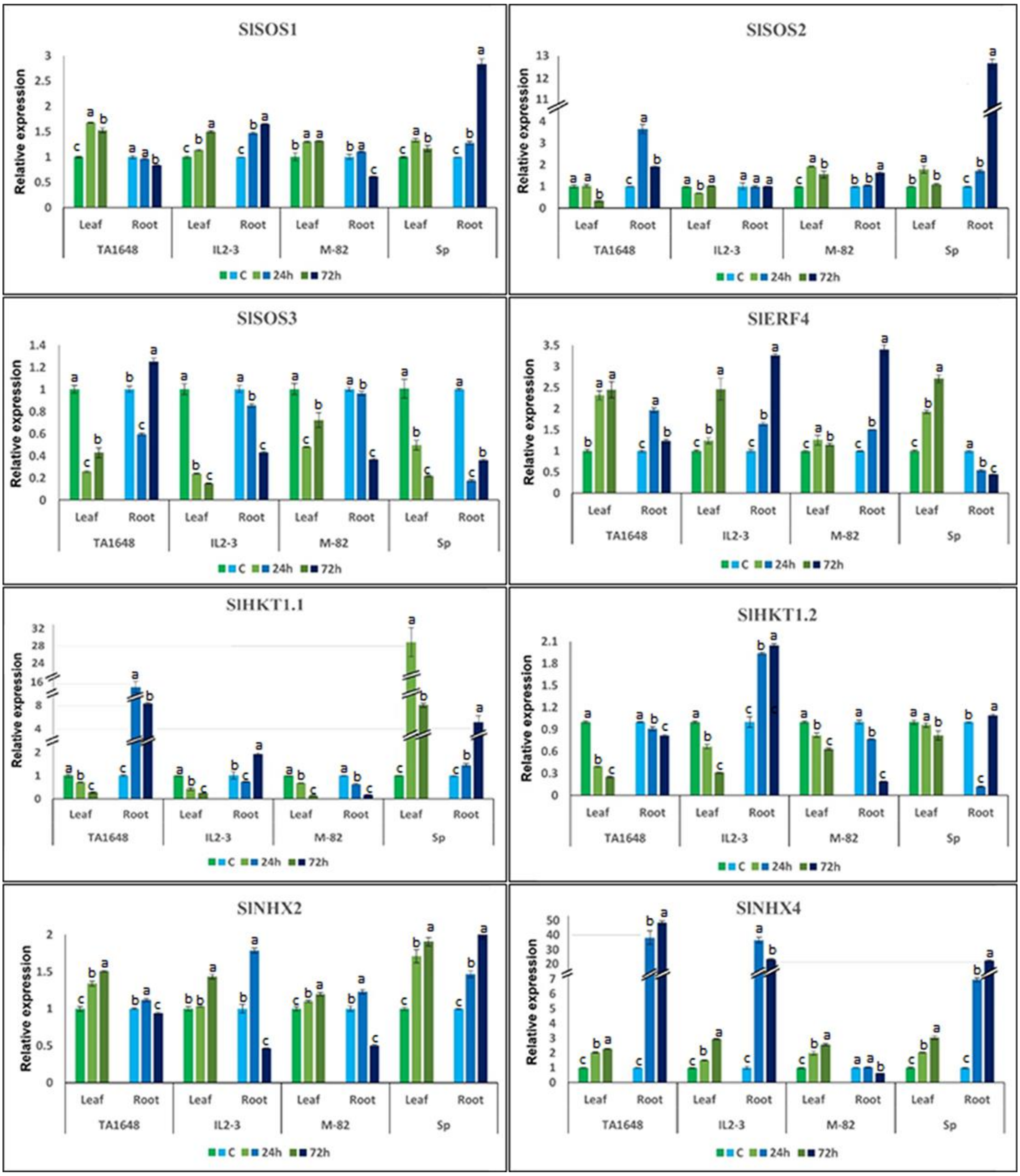

Figure S1. Expression profiles of eight salt stress-related genes in tomato ILs and its relative parents in response to 120mM NaCl after 24 and 72 hours of treatment. Data from qRT-PCR experiments were analyzed according to the 2−ΔΔCt method. The housekeeping actin gene was used as an internal reference control to normalize the expression levels of the target genes. Vertical bars indicate standard deviation calculated from three replicates. Values are mean ± SD. (n = 3) at $p < 0.05$.
